# Sustainable adoption of noninvasive telemonitoring for chronic heart failure: A qualitative study in the Netherlands

**DOI:** 10.1177/20552076231196998

**Published:** 2023-08-28

**Authors:** Stefan L. Auener, Simone A. van Dulmen, R van Kimmenade, Gert P Westert, Patrick PJ Jeurissen

**Affiliations:** 1 Scientific Center for Quality of Healthcare (IQ healthcare), Radboud Institute for Health Sciences, 213763 Radboud University Medical Center, Nijmegen, The Netherlands; 2 Department of Cardiology, 213763 Radboud University Medical Center, Nijmegen, The Netherlands

**Keywords:** Heart failure, telemedicine, telemonitoring, remote monitoring, implementation science

## Abstract

**Objective:**

Noninvasive telemonitoring aims to improve healthcare for patients with chronic heart failure (HF) by reducing hospitalizations and improving patient experiences. Yet, sustainable adoption seems to be limited. Therefore, the goal of our study is to gain insight in the processes that support sustainable adoption of telemonitoring for patients with HF.

**Methods:**

We conducted semi-structured interviews with 25 stakeholders that were involved with the adoption of telemonitoring, such as healthcare professionals, policymakers and healthcare insurers. We analyzed the interviews by using a combination of open-coding and the themes of the Non-adoption or Abandonment of technology by individuals and difficulties achieving Scale-up, Spread and Sustainability framework.

**Results:**

We found that telemonitoring projects have moved beyond initial pilot phases despite a high level of complexity on multiple topics. The patient selection, the business case, the evidence, the aims of telemonitoring, integration of telemonitoring in the care pathway, reimbursement, and future centralization were items that yielded different and sometimes contradictory opinions.

**Conclusions:**

This study showed that the sustainable adoption of telemonitoring for HF is a complex endeavor. Different aims and perspectives play an important role in the patient selection, design, evaluations and envisioned futures of telemonitoring. High conviction among participants of the added value that telemonitoring may support further adoption of telemonitoring. Structural evaluations will be needed to guide cyclical improvement and adapt programs to employ telemonitoring in such a manner that it contributes to collectively supported aims.

## Introduction

Chronic heart failure (HF) is a highly prevalent chronic disease and many developed countries spend approximately 1% of their healthcare budgets on HF associated health care costs.^1^ In 2017, it was estimated that more than 64 million patients suffer this disease worldwide.^2^ The prevalence of HF continues to rise due to the ageing populations and increased survival rates. Simultaneously, healthcare budgets and personnel are overstretched and overloaded, stressing the need for preventing additional costs and workload. Noninvasive telemonitoring, hereafter telemonitoring, may present an opportunity, as it may improve healthcare outcomes and patients’ experiences while reducing healthcare utilization.^3^ Telemonitoring knows a variety of definitions but it generally involves “the remote monitoring and transfer of physical parameters”.^4^ Reports on telemonitoring as a mean to reduce hospital admissions in HF were published as early as 1999.^5^ Yet, the adoption of telemonitoring for HF seems to be slow in many developed countries.^6–8^

Adoption of innovation is a complex and continuous process. This process starts the moment decision makers are aware of the intervention and is followed by several adoption phases such as ex-ante evaluation, implementation, and acceptance until the innovation is in full use.^9,10^ Previous studies have identified many barriers and facilitators. These studies found that factors such as patient characteristics and needs, a lack of funding, organizational characteristics and technical issues affect the adoption.^9–14^ Many of these barriers and facilitators are universal as they occur in many countries.^14^ Another important factor for lagging implementation is a lack of conclusive evidence. There has been inconsistency in reports on important outcomes such as hospitalizations and outpatient visits,^15^ which may be due to factors such as adherence and patient selection.^16,17^ Despite the inconsistency between individual studies, a recent meta-analysis showed that telemonitoring can deliver on reducing mortality and HF-related hospitalization survival.^18^

One of the major drawbacks of telemonitoring is that it may potentially increase the workload of healthcare professionals due to the time needed for following-up alarms^8^ and additional visits.^15^ This stresses the need to adopt telemonitoring in such a manner that it not only improves health outcomes but also supports efficient healthcare delivery. This will be essential for widespread and sustained use of telemonitoring programs that add value for the patient, the provider and healthcare system, which we define as sustainable adoption. The effective use of telemonitoring requires a comprehensive understanding of the underlying processes during implementation. However, studies exploring the barriers and facilitators rarely provide insight in these underlying processes.

Despite the presence of many barriers and knowledge hiatus, there has been uptake of telemonitoring for HF among medical specialists in the Netherlands.^8^ Moreover, use of e-health in cardiology increased significantly due to the COVID-19 crisis.^19^ This means that an increased number of healthcare provider has gained experience with the implementation of telemonitoring beyond pilots and randomized trials. The transition from small-scale piloting to fully-scaled operations and embedding gives rise to new experiences. As telemonitoring moves beyond small-scale pilots, new interests and perspectives emerge from stakeholders such as policymakers and healthcare insurers. Empirical research of telemonitoring projects beyond initial adoption phases is scarce.^11^ The aim of this study is to draw on these empirical experiences and perspectives to create new insights into the factors and processes that support the sustainable adoption of telemonitoring for HF.

## Methods

### Setting

This study was situated in the Dutch healthcare system which is characterized by its model of managed competition in which both healthcare insurers and providers are private, yet, mostly non-profit organizations.^20^ The basic healthcare insurance package, which covers most care after a deductible of 385 euro, is obligatory for residents in the Netherlands.

Software of telemonitoring programs is purchased by the healthcare provider from suppliers or developed by the hospitals themselves. While telemonitoring programs within the Netherlands differ, they generally involve the monitoring and transfer of physiological data. Additionally, some telemonitoring programs include an educational component. The last few years, there have been several developments in the reimbursement options for telemonitoring, these are annually published by the Dutch Healthcare Authority.^21^ It is important to note that in general there are no costs for patients to receive telemonitoring but that for example, smartphones, tablets or weighing scales are not always provided by the hospital.

HF in the Netherlands is traditionally treated in the hospital, of which a majority developed specialized HF clinics. These HF clinics differ both in size as well as staff. Treatment consists of a multidisciplinary approach based on national and international guidelines.^22,23^ More recently, a national program was developed to support transmural collaboration throughout patient journeys and referral to the general practitioner when the patient is clinically stable.^24,25^

### Theoretical framework

**Figure 1.**
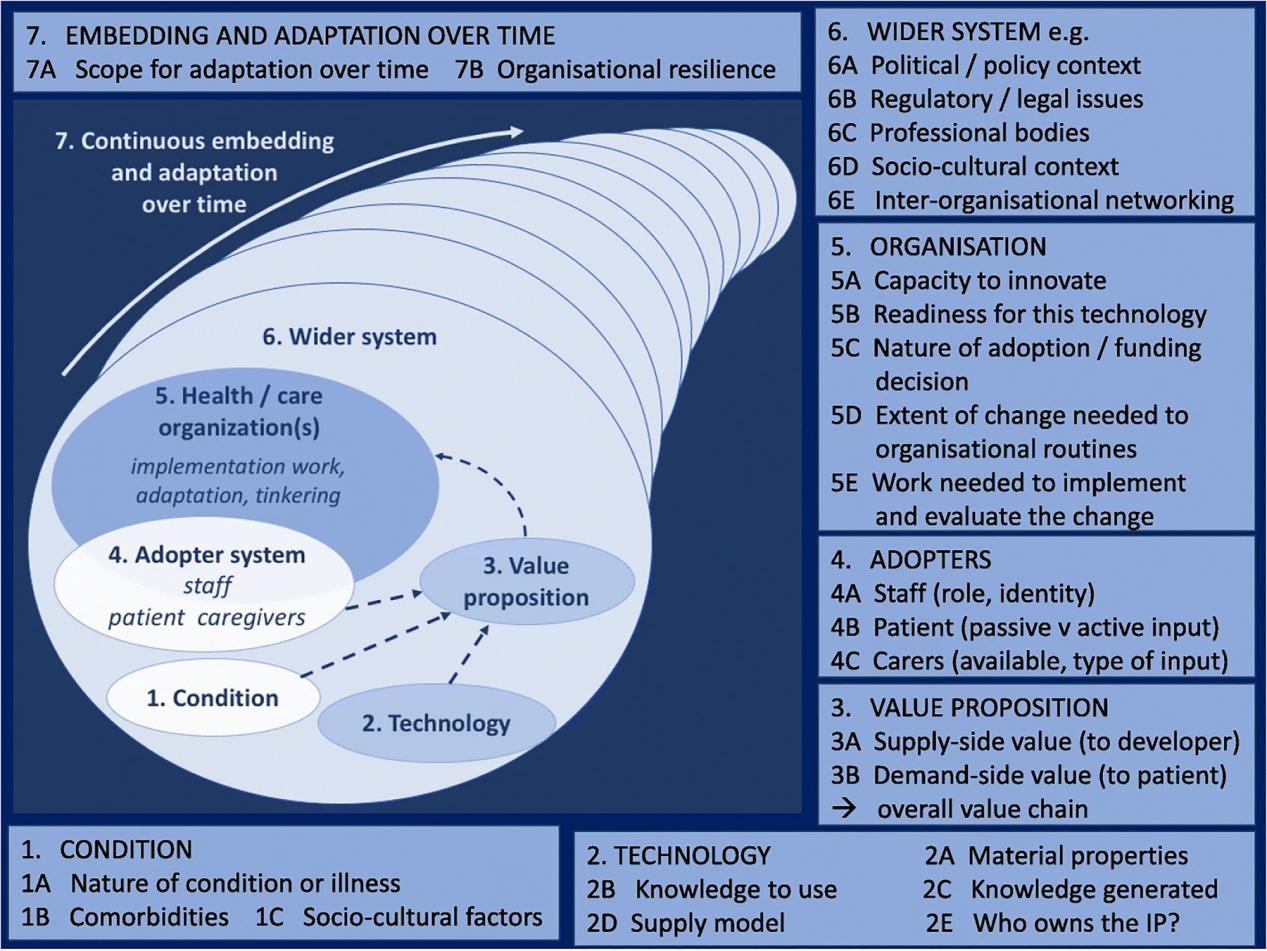
The themes of the NASSS framework. Source: Ref. 26, used under Creative Commons CC-BY license (http://creativecommons.org/licenses/by/4.0/).

We conducted semi-structured interviews among stakeholders involved with the adoption of telemonitoring for HF patients. We used the Non-adoption or Abandonment of technology by individuals and difficulties achieving Scale-up, Spread and Sustainability (NASSS) Framework from Greenhalgh et al. to develop our interview guide and analyze interview transcripts.^26^ This framework consists of seven themes and was specifically developed to gain understanding of processes that affect the adoption towards the sustained use of technologies. Figure 1 shows the themes of the NASSS Framework.

This framework offers a broader scope than frameworks such as the Technology Acceptance Model which do not incorporate the wider system.^27^ The NASSS framework is also more directed at technological innovations than frameworks aimed at general innovation and implementation such as the Measurement Instrument for Determinants of Innovations framework^28^ and Consolidate Framework for Implementation Research.^29^ Another important characteristic is its incorporation of interaction between different domains and how this might affect the adoption. The NASSS framework does explicitly not aim to result in a complete list of barriers and facilitators, but rather to guide conversations and gain understanding of adoption processes.^30^ This resembles the goal of this study.

### Ethical considerations

The study protocol was assessed by The Medical Ethics Committee of the Radboud University Medical Centre which determined that this study was not subject to the Dutch Medical Research Involving Human Subjects Act (registration number 2020-6164). Therefore, ethical approval was not needed. This study complied with the Declaration of Helsinki.

### Participants

Participants were selected through heterogenous sampling, a form of purposive sampling, which is used to achieve a greater understanding from multiple angles.^31^ In addition, we used snowballing to recruit additional participants. We aimed to invite stakeholders with extensive knowledge and experience on the subject of telemonitoring adoption from a variety of perspectives. Therefore, we invited stakeholders that were closely involved within single site telemonitoring projects for HF such as project leaders and cardiologists, but also stakeholders that were involved in multiple telemonitoring projects such as healthcare insurers, umbrella organizations and government officials. Participants were invited by e-mail and provided written informed consent prior to the interview.

### Interviews

Interviews were conducted and recorded through videocalls with Zoom and Microsoft Teams between July 2020 and August 2022. All interviews were conducted by the author SLA (male) or SvD (female), or both, until data saturation was achieved. Both interviewers had been trained in qualitative research and performed qualitative research before. As several respondents were interviewed during the beginning of the COVID-19 crisis and remained intensively involved with telemonitoring throughout the study horizon, we invited two participants for an additional interview at the end of the study period to gain additional insight in the developments throughout the COVID-19 pandemic. The generic interview guide is shown in Appendix 1 and was based on the themes of the NASSS-framework. In preparation and during each interview, additional in-depth questions were asked to gain a deeper understanding of specific subjects. Questions of themes that were outside the area of expertise of the participant were omitted.

### Data analysis

Interviews were transcribed ad verbatim. Thereafter, we performed thematic analysis in ATLAS.ti by using a combination of open-coding and the themes of the NASSS framework. Each code consisted of a prefix number, referring to the relevant themes of the framework, and an open code, describing the content of the phrase. In case there was no clear indication to which theme the phrase was most associated with, the code was assigned a question mark as prefix. The first three interviews were coded independently by two researchers. Subsequently, coded interviews were discussed and checked for inconsistency. In the case of inconsistency, the code was discussed until agreement had been reached. Thereafter, subsequent interviews were coded by SA and five additional interviews were checked by SvD. After all interviews were analyzed, codes were exported to Microsoft Excel for axial coding. Axial coding was performed per theme and codes which were not assigned a theme previously were then classified. Subthemes and supporting codes were discussed over the course of three meetings by SA and SvD. The study is reported according to the COnsolidated criteria for REporting Qualitative studies checklist which is provided in Appendix 2.^32^

## Results

A total of 27 stakeholders were approached through e-mail for an interview. We conducted interviews with 25 participants. Two invitations were declined due to a lack of time. Table 1 shows the participants role and employer. The duration of an interview was on average 57 min and ranged from 29 to 86 min.

**Table 1.** Participants’ organizations.

Organization	Number of participants
Government officials (policymakers, inspector)	3
Healthcare insurers (purchasers, policymakers, advisors)	8
Hospitals (cardiologists, project leaders, advisors)	10
Patient and professional associations	3
Telemonitoring supplier	1
Total	25

Participants that were employed by hospitals were employed by seven different hospitals that were different in terms of size and geographical organization. Exact numbers on the use of telemonitoring for HF were not always available. However, participants reported that it was embedded within their care pathways and beyond the pilot phase. Moreover, telemonitoring was often applied for multiple chronic diseases in the hospital such as high blood pressure and COPD. The telemonitoring supplier we interviewed was one of the major suppliers in the Netherlands. Participants employed by healthcare insurers were employed by three different major healthcare insurers.

Below, we present our results according to the domains of the NASSS framework. Some domains have been separated in sub-themes. Quotes have been collected from the interviews to support and illustrate our findings.

### Domain 1: Illness

Many participants mentioned that the high prevalence of HF played an important role in the decision to look towards telemonitoring for HF specifically. While many participants reported that there were very few barriers for patients to use telemonitoring, opinions differed which patients should receive telemonitoring. This also depended on the aims of the telemonitoring program (Domain 3: value proposition). Some healthcare providers suggested that primarily unstable patients should receive telemonitoring as these were at high risk of being hospitalized. Other participants suggested that almost all patients with HF could benefit from telemonitoring as a tool to facilitate self-management and patient-education and promote a feeling of being safe.

*“So the New York Heart Association stage of the condition also plays a role in this and I think that these are all things that have only been partially, and not yet sufficiently explored. Because you don’t just want to know where it works, but also in which patients you shouldn’t do it.” -Cardiologist*

### Domain 2: Technology

Whereas some hospitals developed telemonitoring programs themselves, others outsourced this or used an external provider. Important reasons for choosing an external provider were that they had more expertise and were more flexible regarding software changes. Important requirements mentioned by participants when looking for an external telemonitoring provider were: a large company to ensure continued support, willingness to co-develop telemonitoring programs, user-friendliness of the software, costs, and the number of conditions supported by the supplier. In addition, medical specialists often played an important role in the decision which supplier was chosen. In some hospitals, there was a preferred provider, selected by a steering board, for which funding and technological aspects were facilitated by the hospital. A few participants mentioned that selection processes of external providers, despite improving over time, still were often unstructured and largely based on experiences of colleagues.

*“but they have determined as a hospital: [supplier’s name], unless. That is a clear policy. That if you have an idea or want to apply something or implement something as a specialist, then you know, okay, I have to see if [supplier] can offer that and otherwise I have to take a slightly different path and it will be more difficult.” -Advisor hospital*

In general, participants noted that current telemonitoring programs were easy to use for both patients and healthcare providers. The barrier most frequently mentioned from a technology perspective was the integration of the telemonitoring program with electronic health record systems. While there were mitigating options such as front-end integration and the use of intermediate platforms that integrated multiple e-health programs, the process was described as cumbersome.

Other suggested improvements for the technology aspect of telemonitoring were to incorporate automated feedback on measures of physiological parameters and lifestyle, and to include noninvasive measurements of left ventricular pressure and arrhythmia. In addition, several participants mentioned that advanced software through artificial intelligence may enable to decrease the number of false alarms by using threshold based on individual variation instead of static thresholds. Improvements of the application were often incorporated gradually over time through regular evaluations with healthcare providers and patients.

*“I think we’ve updated the app like 50 times now. So every time a nurse or a patient came up with a suggestion, we first discussed it in the weekly meetings. Subsequently biweekly. Now we do that once a month. And yes, we are making changes to the program. This can be, for example, a cut-off value, at which the alarm goes off. We have also made the cut-off values increasingly smarter.” -Cardiologist*

### Domain 3: Value proposition

Within the theme value proposition, three subthemes emerged: the aims of telemonitoring, perspective on evidence, and the business case of telemonitoring. These subthemes were strongly connected with subthemes from other domains. Furthermore, the aims of the telemonitoring program affected the configuration and setting of the program, which consequently affected the business case.

#### Aims of telemonitoring programs

Participants often mentioned that the telemonitoring programs aimed to support self-management and reduce inpatient healthcare utilization either through prevention of exacerbations by early detection, or a decrease in the number of outpatient visits. Other positive results were an increase of time for patients when they need care, shortened outpatient visit as measurement were readily available, improved work experience, a reduction of carbon emission and travel time, increased insight in the disease trajectory due to continuity of data for both patients and healthcare providers, increased patient satisfaction, feeling of safety for the patient, shortening the titration period, managing more patients with the same number of healthcare personal, decreased waiting times, and decreased societal costs as patients take less time off work.

Evaluations of the primary aims differed, and were at the time of the interviews limited to business analyses as none of the participants published their findings in a peer-reviewed publication. Most interviewed participants with telemonitoring programs mentioned the ambition to perform enhanced evaluations on the effect of healthcare utilization in the near future. They also pointed towards barriers such as a lack of in-house methodological capacity in performing such research, extracting data from EHRs, and the confounding effect of COVID-19 and other factors such as new medication being available. Evaluation of different telemonitoring programs was also a high priority for healthcare insurers which were frequently part of steering groups. Even when initial targets were not achieved in the short term or could not be evaluated, several participants stressed the importance of the opportunity to learn together in practice.

*“The extent to which hospitals are really evaluating different outcomes varies greatly. And I think that also has a lot to do with the primary goal, so to speak. Your approach could be that you want to give the patient more control or, say, you want to deal with capacity problems or you want to coach the patient much more in what he is doing. These are all completely different approaches where completely different outcomes are relevant.” -Government official*

#### Perspective on evidence

We found that there were different paradigms on how to look at telemonitoring as an intervention which in some cases caused friction on which evidence is needed. As some participants perceived telemonitoring as digitalized traditional care, thus the same care in another way, high satisfaction among patients should be enough reason to adopt this technology. Others deemed telemonitoring a new intervention which needs to prove its effectiveness before adoption. The need for a shared vision on evidence was also mentioned several times. Some participants found that traditional Randomized controlled trials (RCTs) were not deemed fit for the evaluation of complex interventions such as telemonitoring. Others deemed RCTs more essential for assessing the effect of telemonitoring. All interviewed participants acknowledged the complex and heterogenous reality of telemonitoring, and stressed the need for taking the context and settings of the telemonitoring program into account when evaluating these programs.

#### Business case

The business case, referring to the investments needed and the benefits pursued, was often mentioned as a complex matter. This was largely contributable to business cases that were different for the healthcare provider and the healthcare insurer. From a hospital perspective, it was hard to have a positive business case as the hospital invests in a telemonitoring infrastructure that reduces healthcare utilization and thereby revenue under traditional payment models which incentivize production. Participants mentioned that costs from hospitals are for more than 80% fixed costs that are hard to adapt to declining revenue. Whereas some participants noted that increasing prevalence of HF mitigates a declining revenue, others participants mentioned that the decline of revenue was too quick to be mitigated by a slow increase of prevalence. From a healthcare insurer perspective, participants noted that a theoretical business case may be positive but that collecting in on monetary benefits is very hard as healthcare providers often provide other reimbursed services when certain care is reduced or eliminated.

Major determinants affecting the business case were the intended population (Domain 1), licensing model used by the supplier (Domain 2), integration in care pathways (Domain 5), and payment models (Domain 6). Licensing models available from different suppliers ranged from fixed costs per patient to one license to cover all use within one hospital over transcending departments resulting in different business case results in combination with patient selection. Most participants mentioned that a smart integration of telemonitoring in the care pathway was essential to be successful as a wrong deployment of telemonitoring could even cause additional workload. All participants mentioned that the business case should not be limited to monetary effects but should also include others benefits of telemonitoring. Participants mentioned benefits such as reduced travel time, increased insight in disease trajectory, and self-management.

### Domain 4: Users

This domain refers to how patients’ and healthcare professionals are affected by the adoption of the innovation and what is expected from them. While we did not interview HF patients directly, most participants mentioned that own evaluations showed good results on patient satisfaction. In addition, several participants reported that the satisfaction of telemonitoring has a strong evidence base in scientific literature. The main reason for patients to abandon telemonitoring is a mismatch with personal characteristics or preferences or a lack of digital skills.

*“Sometimes people are not digitally skilled enough. There are really people over 80 years who use the app and who don’t find it a problem, but for some people it’s still too difficult. On the other hand, you also have people who are technically able, but who get an obsession with continuous measurement because of the app, who then measure their blood pressure every half hour. Then you are also not suitable, but on a different basis.” -Project leader hospital*

Several participants noted that telemonitoring also requires more involvement of patients with their own health to increase self-management as just the process of transmitting values on itself does not support self-management. Several participants mentioned that patients should be enabled to demand telemonitoring as this may be a strong facilitator for further adoption.

*“Once you give them the blood pressure monitor and you have to press a button and it is transferred and then you loosen that strap again, that is a little less confrontational than if you enter those numbers yourself twice a week. Then, of course, the patient also sees that trend.” -Cardiologist*

Most participants noted that there is high variety among healthcare professionals in the willingness to use telemonitoring and the trust of healthcare providers in technology. Fears of healthcare professionals were mostly related to increased workloads due to the checking of transmitted values. In addition, healthcare professionals considered direct contact with their patients important and feared this was reduced by deploying telemonitoring. Participants suggested that representatives for different healthcare professions should be closely involved with the implementation process and organization to ensure support. One participant noted that there was high interest from nurses for working in a hospital central telemonitoring center as there were no night shifts for nurses and telemonitoring was perceived attractive as it has an image of being highly innovative.

### Domain 5: Organization

We found multiple subthemes on the organizational level that could affect the adoption of telemonitoring. These consisted of characteristics of healthcare providers but also the way telemonitoring was adopted within organizational routines and how data was processed.

#### Organizational characteristics

Participants suggested there may be several characteristics that act as facilitators for hospitals. These facilitators were having a large enough number of patients to enjoy scale benefits, enough resources for hospital-wide facilities such as the presence of a chief medical information officer, a steering group, a collectively paid central monitoring department, willingness to outsource parts of the telemonitoring process, or plans for a new hospital building in the near term which allows for reducing fixed costs. While some participants mentioned that university medical centers were slower with adoption than the large hospitals possibly due to a ‘not invented here syndrome’ or a desire to keep researching, others did not recognize this. Two participants noted that there is very high heterogeneity in HF clinics in the Netherlands in terms of organization, number and kind of healthcare professionals and the HF populations. Moreover, they mentioned that HF clinics from university medical centers are often specialized in specific populations in which telemonitoring may be more or less useful.

#### Shared strategy

An important facilitator for the implementation of telemonitoring was a strategy that was supported by both the medical profession and the board of directors. Most participants estimated that a bottom-up approach was more likely to be successful as it is essential that there is support from healthcare professionals. However, simultaneously, the board of directors does need to be vocal about the hospital's strategy and mission, and instruct departments to contribute to this mission.

*“You really have to show as a hospital that you are also in favor of digitalization of healthcare. Therefore, you not only have to facilitate it well, but you also have to instruct the departments to contribute to this aim. So, we want to implement a piece of digitalization to ultimately make it cheaper, what are you going to contribute to that?” -Advisor hospital*

#### Care pathway

Almost all participants found the way telemonitoring is integrated within the organization of care extremely important to be successful. This was often referred to as developing the right care pathway and the vast majority of participants stated that this was often overlooked. It was acknowledged that this process took significant time but was critical. Most participants stated that a failure to integrate telemonitoring severely reduced the potential benefit and can even increase the workload of healthcare professionals.

Several participants noted that hospitals often started with small number of patients to explore the use and effect of telemonitoring without substitution of parts of the traditional care pathway. They reported that this method of implementation can increase support as healthcare professionals can slowly get adjusted. However, it also created a potential pitfall when the organization reaches a tipping point during the scaling up and changes are needed in the care pathway. Several participants noted that it was important to start with the development of the care pathway and only afterwards select the technology. The way telemonitoring was integrated in the care pathways differed among the interviewed healthcare providers but a common denominator was the use of telemonitoring as substitution for outpatient visits, or to facilitate a shift from more intensive care to lower intense care.

*“And the crux is that you do not do it alongside existing care, but that you actually work on a transformation of the way in which you have organized the care. And if you do it in addition of usual care with small numbers in a pilot and you don’t adjust your care path and process and you don’t stop doing other things, then it’s just completely unprofitable.” -Policy advisor government*

To adjust the telemonitoring program to patient needs and to support efficient use of telemonitoring, several healthcare providers reported that they had specific protocols for different patients. In these protocols, the patients that were recently hospitalized, or started titration, had measurements more frequently than patients that had been stable for a certain period of time. Titration, the adjusting of medication to achieve optimal medical treatment, was mentioned multiple times as a period in which telemonitoring added major benefit. Participants reported that traditional titration required frequent outpatient visits to check on physiological parameters and that many of these visits can be substituted by telemonitoring. This enables a reduction in the number of outpatient visits. Moreover, due to the additional measurements, it reduces the time needed to reach optimal medical treatment doses.

One side effect of telemonitoring which was mentioned a few times was that as telemonitoring substitutes outpatient visits, there will be an increase of the average complexity of the patients visiting the HF clinic. Participants mentioned that the change in case-mix should be accounted for in the organization of the HF clinic and the funding system (Domain 6).

#### Processing data

Besides adapting the care pathway, the telemonitoring program often required a structured way to process incoming measurements. Most participants mentioned that improvement of algorithms should reduce the number of false-positive alarms. Some hospitals outsourced part of the processing of data to external parties. Other participants reported that they processed the telemonitoring data within the department or centralized this process within the hospital in a dedicated telemonitoring department. Opinions differed strongly between participants regarding the degree to which alarms generated by telemonitoring should be processed centrally. This ranged from the telemonitoring program being managed within the department, to a centralized telemonitoring department within the hospital, to regional or even national centers. Primary reasons for being more in favor of a more decentral or a central approach were maintaining a direct relationship between the patient and effectiveness in processing data, respectively.

*“I see more need for improving the algorithms in order to make an efficiency boost than to simply place that in the hands of large centers. On the other hand, we are not that far at the moment, so you could also say, outsource now and then alarms will come in and they will be passed on to the relevant care providers. It may be an interim solution, but in the end I wonder whether this can really improve efficiency or whether it is ultimately an extension of care.” -Cardiologist*

*“And well, we became part of the team. And that is precisely the reason why we do not believe in seven national monitoring centers, because then you go too far from the care for our own patients. We have become part of a treatment team of our patients, and we do not believe that if you organize that nationally, you will do it just as well. And we even think that the effectiveness… It does become more efficient for the monitoring center, but it becomes less efficient for the specialism because they actually no longer know the colleague.” -Project leader hospital*

*“..because the danger of this is, at least I see that, is that each hospital is going to set up its own data center and that's not going to be effective. So, I very much believe that we are moving towards a setting where you have one, two, three, four data centers in the Netherlands and that you can then, so to speak, serve all patients from there.” -Employee healthcare insurer*

### Domain 6: Wider system

Participants experienced several barriers embedded within the healthcare system that may hinder structural embedding of telemonitoring in regular care processes.

#### Funding

A major subtheme was funding, which is closely related to the business case in Domain 3. Whereas the business case refers to the costs and benefits for a telemonitoring program from different perspectives, funding relates to the ways these costs and benefits can be transferred among stakeholders. Most participants mentioned that pilots with limited number of patients and a temporary character often do not need additional funding but that embedding within regular care introduces issues as costs and benefits become structural. As several telemonitoring programs had hundreds of patients on telemonitoring, this was indeed raised as concern several times. There were several payment models between healthcare insurers and healthcare providers. Participants reported bilateral multi-year lump sum agreements which aimed to support hospital-wide transformation, labeled funds for specific transformation projects, and bilateral agreements for per patient reimbursement. In addition, there was a universal care product for telemonitoring which was yet to be introduced at the time of the interviews. Opinions differed whether these payment models allow for adequate financing of telemonitoring as each payment model had it arguments for and against. However, the vast majority of participants reported that, without adjustment of traditional funding, deploying telemonitoring is not financially attractive for hospitals.

*“It is known that the costs are going down, but the revenues are going down even harder. Yes, that is not immediately something that a hospital organization is very interested in. When it comes to small numbers, it’s fine, but if you’re scaling up, you run into that, so you have to do something with that.” -Employee of Professional Association*

Besides reimbursement from healthcare insurers, the internal revenue distribution models within hospitals also brought challenges as costs and benefits may not be attributable to the same department. Participants mentioned that these distribution models need to be in line with the goals pursued. As an aim of telemonitoring may be the reduction of expensive healthcare utilization, this may lead to decreased revenues for departments. Several participants therefore suggested to introduce other measures in the distribution model that incentivize behavior that support other pursued goals, such as shortening waiting lists and improving quality of care.

*“The moment you agree on a lump sum agreement with the hospital, you also have to adjust agreements about revenue distribution within the hospital. Because it doesn’t make much sense if the departments’ revenues are still driven by production and the hospital now on a different model.” -Employee of healthcare insurer*

#### External parties supporting telemonitoring

Several participants mentioned that some healthcare professionals may be wary of the legal consequences and responsibilities and were therefore hesitant to start telemonitoring. However, most participants mentioned that, while especially the construction of data processing agreements with external parties required some time, there were no legal objections which prevented the implementation of telemonitoring. To inform healthcare providers on the legal requirements, the Dutch healthcare inspection developed an assessment framework based on current laws and regulations.^33^

A facilitating factor was that many parties such as different governmental organizations and umbrella organizations facilitated the dissemination of experiences and information. Moreover, healthcare insurers and telemonitoring suppliers supported implementation and evaluation by providing insights in the use of telemonitoring in other hospitals, e.g. use of thresholds values for parameters. In addition, the Dutch healthcare authority developed reimbursement options to accommodate to the needs by healthcare providers and healthcare insurers.

### Domain 7: Time

Domain seven refers to the embedding and adaptations over time that lead to and affect the sustainability of telemonitoring programs. Interviews were conducted throughout the COVID-19 pandemic, which was also mentioned as important theme affecting adoption. Many participants noted that healthcare providers and patients both gained digital experience and several participants labeled the COVID-19 crisis as a true eye-opener which gave a boost to digital care. Participants were unsure if this boost would sustain in the foreseeable future as some healthcare providers and patients may value face-to-face contact even more after being obliged to work digitally after a prolonged period during the COVID-19 crisis.

Several participants noted that regulatory changes were introduced by the Dutch healthcare authority made in response to COVID-19, and that cooperation between parties such as healthcare insurers and healthcare providers was easier compared to prior COVID-19.

Several participants envisioned a telemonitoring program in the near future in which the general practitioner was also closely involved and patients stay on telemonitoring after being referred to the general practitioner. These participants also noted that such a development will also be likely to encounter different barriers such as funding and additional difficulties regarding the communication and sharing of patient data among healthcare providers.

A vast majority of participants explicitly mentioned that, while improvements are needed and a collective learning processes need to be continued, they were convinced of the added value of telemonitoring in the future.

## Discussion

### Principal findings

The aim of this study was to provide insight in the processes that affect sustainable adoption of telemonitoring for HF in the Netherlands. More and more healthcare providers have moved beyond initial pilot phases. Almost all participants were convinced that telemonitoring can add value to the care for patients with HF. However, we also found that participants hold different, and often multiple aims. These aims affect the preferences regarding the deployment of telemonitoring. The patient selection, the business case, the evidence, the aims, integration of telemonitoring in the care pathway, and reimbursement were items that yielded different and sometimes contradictory opinions.

Moreover, we witnessed interdependence between the different domains from the NASSS framework, which drove complexity of barriers. The challenges for implementation and structural embedment of telemonitoring do not stem from individual barriers but rather from the interdependency between the many components. This is in line with the theory behind complex systems, and as proposed by Greenhalgh et al., the creators of the NASSS framework.^34,35^

### Reflection on our findings from a practical perspective

We found many influencing factors that have been previously reported in scientific literature such as integration of software, patient and professional preferences, and lack of reimbursement.^11,14^ Several of our findings have received limited attention in scientific literature, yet, provide important insights in processes that affect sustainable adoption of telemonitoring for HF. These were: (a) the presence of multiple aims for telemonitoring; (b) the use of tailored telemonitoring programs; (c) the integration of telemonitoring as an iterative process; and (d) the evaluation of telemonitoring programs.

#### The presence of multiple aims for deploying telemonitoring

The European Society of Cardiology guidelines describe the primary goals of treatment for HF which consist of reducing mortality, reducing hospitalizations and improving clinical status, functional capacity and quality of life.^22^ We found that many participants pursued additional aims with telemonitoring for HF such as more efficient use of healthcare personal and improving patient experiences. Moreover, some of the aims pursued by participants, such as increased self-management have not been validated by scientific studies yet.

The NASSS framework proposes an interaction between the domains which in turn affects the value proposition.^30,35^ We found that in this case, the aim, which is the pursued value proposition, also affects the other domains to a great degree. For example, participants that were opined that the primary aim of telemonitoring was a cost-effective reduction of healthcare utilization, were also more opined that it should be deployed within high-risk populations. Furthermore, they were more likely to be in favor of centralization. On the other side, participants that mentioned supporting self-management as primary aim were more likely to favor limited centralization and less restricted inclusion criteria for patients. This discrepancy is likely due to the multi-purpose nature of telemonitoring as it has been portrayed as a solution for a multitude of challenges. As new complex interventions such as telemonitoring are implemented in clinical practice, involved parties need to be aware of the aims from different perspectives. Therefore, they should discuss the interactions between the aims of the involved stakeholder to establish a shared strategy. Failure to do so may result in the loss of support for telemonitoring by stakeholders.

#### Tailored telemonitoring programs

Participants acknowledged the importance to deploy telemonitoring for the right patients using patient selection. Some telemonitoring programs were exclusively offered to patients that were deemed unstable, as this was deemed cost-effective due to the high risk of rehospitalization of this population. Other telemonitoring programs had multiple protocols for different patients to meet different needs. The need for more specific patient selection to maximize expected benefit has been previously identified by Fairbrother et al.^13^ Patient inclusion criteria may be based on clinical characteristics such as New York Heart Association class but also through other indicators of high risk at hospitalization such as biomarkers.^36^ One of the encountered protocols that was referred to multiple times, was aimed at patient undergoing titration (the adjustment of medication to optimal levels). As traditional titration protocols require frequent outpatient visits, participants found that the use of telemonitoring might greatly reduce outpatient visits as telemonitoring makes these visits redundant. In addition, participants mentioned that the time until optimal medical treatment decreased. The added benefit of remote monitoring for titration purposes has also been shown previously in other studies.^37,38^ Increased number of patients on optimal medical treatment has been suggested to be an important added value of telemonitoring to reduce hospitalizations.^39^ The finding that different telemonitoring programs can be tailored for specific needs illustrates that telemonitoring should not be perceived as one intervention, but rather as a collection of interventions, serving different target groups.

#### Iterative integration within regular care

The integration of telemonitoring within the care pathway was deemed crucial for an effective process and embedment of telemonitoring. As many telemonitoring programs started out as a pilot with limited number of patients, a transition towards a larger scale operation was required. This transition often involved a tipping point at which issues or questions occurred regarding the integration of telemonitoring into the care pathway. This included the integration within workflows in line with scientific literature.^14^ In addition, many participants explicitly referred to the substitution that should be pursued in order to gain efficiency and prevent additional workload that was experienced by some participants. The most basic example of substitution was full or partial cancellation of traditional follow-up visits.

Moreover, we found that telemonitoring programs, that were deemed successful from participants’ perspectives, were subject to iterative adaptations. These adaptations concerned both technical aspects, such as the thresholds of alarms, as well as organizational processes, such as the scheduled number of outpatient visits. This need for allowing stakeholders to adapt technology and processes has often been neglected by theoretical frameworks but has been identified as essential for implementation process which is in line with our findings.^30,35^ Whereas Papoutsi et al. suggested that site-specific iterations may be required before clinical testing,^40^ our results suggest that these iterative processes may not reach a steady state at all in the near future as technologies and organizations are still developing and adjusting years after initiation.

#### Evaluation of telemonitoring as a complex intervention

Complex interventions also require more extensive evaluations. Systematic reviews showed different results and predominantly refer to the heterogeneity of interventions as an explanation for different results.^41^ During the current study, we found a myriad of possibilities for the design of telemonitoring programs. These designs did not only differ between sites but also evolved over time. In addition, participants struggled with evaluation of the telemonitoring programs to a variety of practical reasons such as extraction of appropriate data, in-house methodological support, but also due to fundamentally different perspectives on the kind of evidence needed. For example, several participants were content with the promising results found in before–after analyses whereas others were more conservative with estimations and referred to the pitfalls of such evaluations. Different evaluation methods may severely affect the results.^39,42^ These different perspectives on the needed evidence can therefore induce gaps in understanding between stakeholders.

Large discrepancies in results show the need for evaluations with more scientific rigorousness. Simultaneously, studies should take the dynamic reality of telemonitoring programs into account. Confounders and context are an integral part of the implementation of complex interventions as they are not implemented in vacuums.^35^ In addition, studies should be more in line with real-world use and developments. While multiple telemonitoring programs we encountered used telemonitoring as a substitute for outpatients visits, the vast majority of trials and observational studies have used telemonitoring as an add-on.^15^ Moreover, many studies on telemonitoring have used “usual care” as comparator but lack extensive descriptions. Usual care for HF is highly heterogeneous across, and even within countries.^43,44^ For example, referral of stable patients to primary care differs across regions.^25^ Stakeholders should be aware that these factors can induce severe uncertainty. Study designs that are better equipped to take these considerations into account are realist evaluation,^45^ RCTs with process evaluation,^46^ platform trials,^47^ and action-based research. The latter allows for the inclusion of cyclical evaluations and adaptions, which already occurred in many telemonitoring programs.^48^

### Adoption despite the presence of complex challenges and the NASSS framework

The NASSS framework provided a structured method for the qualitative evaluation of the developments concerning telemonitoring implementation for HF in the Netherlands. Furthermore, it enabled us to capture the complexity of sustainable adoption which resulted in insights that can further support adoption. Within multiple domains, there were issues that can be classified as complicated or even complex, which does not bode well for adoption.^26^ However, despite the presence of multiple complex challenges, multiple participants reported that they successfully embedded telemonitoring within regular care. This shows that merely having multiple domains with high complexity does not necessarily result in abandonment or a lack of scaling up as proposed by the NASSS framework.^26^

Several facilitators that such as the presence of a clear hospital-wide strategy, clinical championship, and good relationships with healthcare insurers supported adoption. Furthermore, we hypothesize that stakeholders’ belief in the value proposition plays an essential role in adoption in the presence of multiple barriers. While the value proposition may be different and uncertain for each stakeholder, the expected benefit can move stakeholders to invest in this innovation. Meanwhile, adoption can reach a critical point after which telemonitoring is embedded within regular practice. This hypothesis is in line with the Technology Acceptance Model which states that the intention to use is largely affected by the perceived usefulness and perceived ease of use.^27^ Moreover, there is evidence that suggests that perceived usefulness is a better predictor of usage than perceived ease of use.^49,50^ Dyb et al. found that the enthusiasm and dedication of healthcare providers could overcome challenges for technology-supported patient-centered care (PCC). This lead to the conclusion that “*…the point of no return has passed for key healthcare providers. To them, technology-supported PCC is already a definite part of future healthcare services”.*^51^ Dyb et al. also found that after this point, a pragmatic approach was chosen in regard to complex barriers. This is in line with our findings that illustrate such a pragmatic approach such as limited front-end integration of software and continuation of the program even with a currently negative business case. While a common belief in the value proposition can overcome complex barriers, this does not mean that barriers can be ignored. These barriers may still prevent adoption among other professionals who haven’t passed this critical point, and barriers are likely to affect the actual value proposition.

### Future of telemonitoring

The COVID-19 crisis may have provided a burning platform to use this technology. However, major challenges regarding the financial sustainability and shortages of healthcare personal remain. We found two important knowledge hiatus that need to be addressed to support future adoption of telemonitoring. These hiatus concerned the funding model necessary for sustainable adoption and the level of centralization of telemonitoring to improve efficiency.

#### Funding models

Our study revealed conflicting views in regard to appropriate funding models for telemonitoring. This is consistent with literature identifying reimbursement as being one of the most mentioned factors.^9,11^ We encountered different reimbursement schemes to support telemonitoring but there was no clear consensus among participants which scheme should prevail. In addition, there were a lot of recent development and temporary reimbursement options for e-health initiatives similar to other European countries such as French and Germany.^52,53^ The newest funding option in the Netherlands, available from 2023 onwards, was only referred to in the last interviews as it was only recently published.^21^ This option consists of a general claim code that can be used across multiple specialisms. The maximum reimbursement consists of 164 Euro per 120 days and substitution of care is an explicit precondition. This is a different approach to those of other countries like Belgium, which relies more on provisional reimbursement pending scientific evaluation, or Germany, which covers telemonitoring for HF specifically under statutory healthcare insurance as of 2022.^53,54^ While the Dutch approach reduces complexity of funding, it also limits the possibilities for healthcare payers to utilize adaptive pricing for specific indications or telemonitoring programs to stimulate appropriate use and cost-effectiveness.^55^ Future empirical research should evaluate the consequences of these policy decisions.

#### Centralization to improve efficiency

As more and more healthcare providers deploy telemonitoring for a variety of diseases, questions emerge concerning the cost-effectiveness of localized telemonitoring programs for small numbers of patients.^15^ This stresses the need for more insight in the centralization of telemonitoring, which may increase efficiency due to economies of scale. Several clinicians we interviewed were opined that centralization should be limited as external providers do not know the patients and their history. Simultaneously, several employees of healthcare insurers envisioned further centralization through the use of medical service centers. The difference in views may induce friction in the near future as current evidence on centralization options remains scarce. A recent study from Peters et al. in the Netherlands explored potential cost-savings within a program that used telemonitoring to expedite discharge from the hospital.^56^ While this study was targeted at a very specific population, it showed that centralization may deliver on cost-efficiencies. However, it also showed that benefits of centralization diminished above hospital level. This raises the question at which level of centralization sufficient efficiencies are achieved.

Lastly, several participants envisioned a future in which telemonitoring was integrated within transmural collaborative care. Many regions within the Netherlands have developed Regional Transmural Agreements (RTAs) to facilitate such transmural collaboration.^25^ These RTAs are highly heterogenous for HF between regions, which may affect the feasibility and efficiency of different levels of centralization of telemonitoring programs. Future studies should explore the potential synergies and pitfalls of integration of telemonitoring within transmural collaborations and how these relate to the question of centralization.

### Strengths and limitations

A major strength of this study is the inclusion of participants with different backgrounds and a broad scope of the subject by using the NASSS framework. This allowed us to explore a variety of perspectives from different background and how these perspectives interact with the context and value propositions of telemonitoring programs. The inclusion of multiple perspectives also enabled us to identify divergent, and sometimes conflicting, perspectives and interests. As most participants were interviewed during and after the scaling up of telemonitoring programs, our results inform about adoption beyond initial trial experiences.^40^ This lead to an integral overview of processes that play a role in the implementation and future embedment of telemonitoring for HF. Another strength is the high generalizability of our results to telemonitoring programs for other diseases. While some issues we found were disease specific, many of the issues were not. These issues are likely to play significant roles in most telemonitoring programs, independent of the disease or condition.

The main limitation of this study is the selection bias that may have occurred by inviting participants that were closely involved with the adoption of telemonitoring programs. It is likely that these participants have a more positive opinion towards telemonitoring and its merits. Furthermore, we mainly interviewed participants that were involved with the organizational aspects of the implementation of telemonitoring programs and not specific technical aspects which may have introduced under-reporting of such barriers. In addition, we did not include participants that were not directly involved with the implementation or adoption but did use telemonitoring systems, such as healthcare providers and patients. A lack of acceptance can pose a significant barrier for adoption as it can introduce non-adherence, which may not only limit utilization but also the effect of telemonitoring.^17,57^ Such information will therefore also be essential to realize the value proposition of telemonitoring. We deemed that these perspectives should not be forgotten but were out of scope for this study.

## Conclusions

The sustainable adoption of telemonitoring for HF is a complex endeavor. The NASSS framework can be used as analytical tool to gain insight in the processes that underly this complexity. Different aims and perspectives play an important role in the designs, evaluations and envisioned futures of telemonitoring. There was broad consensus that telemonitoring ultimately should act as an integrated tool to support more efficient care pathways. However, integration within regular practice often required prolonged iterative processes. The COVID-19 crisis provided a boost to the adoption. There was high conviction among participants of the added value that telemonitoring ultimately can bring. This will likely support further diffusion of telemonitoring. Structural evaluations will be needed to guide cyclical improvement and adapt programs to employ telemonitoring in such a manner that it contributes to collectively supported aims. In addition, more research is needed to find adequate payment models and gain insight in the optimal embedment of telemonitoring in routine care while taking centralization and transmural collaboration into account.

## Supplemental Material

sj-docx-1-dhj-10.1177_20552076231196998 - Supplemental material for Sustainable adoption of noninvasive telemonitoring for chronic heart failure: A qualitative study in the Netherlands Click here for additional data file. Supplemental material, sj-docx-1-dhj-10.1177_20552076231196998 for Sustainable adoption of noninvasive telemonitoring for chronic heart failure: A qualitative study in the Netherlands by Stefan L. Auener, Simone A. van Dulmen, R van Kimmenade, Gert P Westert and Patrick PJ Jeurissen in DIGITAL HEALTH

sj-docx-2-dhj-10.1177_20552076231196998 - Supplemental material for Sustainable adoption of noninvasive telemonitoring for chronic heart failure: A qualitative study in the Netherlands Click here for additional data file. Supplemental material, sj-docx-2-dhj-10.1177_20552076231196998 for Sustainable adoption of noninvasive telemonitoring for chronic heart failure: A qualitative study in the Netherlands by Stefan L. Auener, Simone A. van Dulmen, R van Kimmenade, Gert P Westert and Patrick PJ Jeurissen in DIGITAL HEALTH
