# Supplementary material for: Sustainable adoption of noninvasive telemonitoring for chronic heart failure: A qualitative study in the Netherlands
Source: Digit Health. 2023 Aug 28;9:20552076231196998. doi: 10.1177/20552076231196998 (PMC10467184; doi:10.1177/20552076231196998)
Supplement: sj-docx-1-dhj-10.1177_20552076231196998 - Supplemental material for Sustainable adoption of noninvasive telemonitoring for chronic heart failure: A qualitative study in the Netherlands [file sj-docx-1-dhj-10.1177_20552076231196998.docx]

Appendix 1: Topic guide

| **NASSS Theme** | **Questions** |
| --- | --- |
| Condition/Ilness | Why was it decided to use telemonitoring in heart failure? i.e. why does heart failure lend itself to telemonitoring? |
|  | In which patients is telemonitoring currently used within the group of heart failure patients? Why were these patients chosen? What other groups within heart failure suitable? (under what conditions/what are the consequences?) |
|  | Which non-medical characteristics of patients are taken into account when considering whether TM is used? How many patients will this affect? |
| Technology | What does telemonitoring in heart failure look like in your hospital? |
|  | How do you process and analyze entered data? What options were there? Why did you choose the current option? |
|  | How do hospitals/do you choose which supplier they/you purchase telemonitoring from? |
|  | How is telemonitoring integrated in existing systems such as the electronic health records? |
| Value Proposition | What is the value proposition according to you? Does telemonitoring deliver on its’ value proposition? |
|  | How do you measure outcomes? |
|  | Which adjustments are necessary to increase the value proposition? |
| Users | How are patients supported? What is expected of patients when using telemonitoring? |
|  | What is expected from healthcare professionals when adopting telemonitoring? How does telemonitoring changes their profession? |
| Organization | Which factors within your organization have supported the adoption of telemonitoring? Which parties within your organization were involved? |
|  | How was telemonitoring integrated within organizational routines? |
| Wider system | Which external factors or parties influence or will influence the adoption of telemonitoring in chronic heart failure? |
|  | How do reimbursement policies and legislation affect adoption? |
| Time | What recent changes have you witnessed?  How do these changes affect the adoption of telemonitoring?  Which changes will be essential for sustainable adoption? |
